# New lumefantrine self nanoemulsifying delivery system – will it by-pass lymphatic transport mechanisms?

**DOI:** 10.1186/2008-2231-21-55

**Published:** 2013-07-08

**Authors:** Nuggehally R Srinivas

**Affiliations:** 1 Suramus Biopharm, JP Nagar, Bangalore, India

## Dear editor-in-chief

The recently published article in the esteemed journal entitled “Design and evaluation of Lumefantrine – Oleic acid self nanoemulsifying ionic complex for enhanced dissolution” [1], describes an in-depth research pertaining to the formulation attributes of lumefantrine, an important ani-malarial drug, to enhance its in vitro dissolution and thereby improving its bioavailability upon oral administration. As discussed by authors’ poor solubility of novel chemical entities (NCEs) leads to lack of in vivo translation of the promising in vitro attributes of NCEs because they fail to achieve adequate exposure needed to modulate the target or receptors under in vivo conditions [1]. Since lumefantrine has a low and variable bioavailability dependent on the food intake, it is extremely challenging to maintain the threshold levels of the drug needed to combat malarial infections [2]. While the dosing recommendation of lumefantrine takes into consideration food intake especially milk consumption to improve its solubility and bioavailability, it may be difficult to maintain the same food intake conditions within the same individual or across individuals who are suffering from malaria. Therefore, the need of a formulation strategy to overcome this barrier and maximize the delivery of lumefantrine was warranted. In this context, the development and complete characterization of a self nanoemulsifying delivery system is the step in the right direction as it potentially eliminates the influence of food on the bioavailability of lumefantrine [1].

In this interesting work, a complete optimization of the nanoemulsifying delivery system has been achieved – consideration has been given to the various excipients and the composition needed to maximize the dissolution of lumefantrine [1]. Interestingly, comparative dissolution profiles of the current marketed formulation vs the newly developed lumefantrine nanoemulsifying delivery system, unequivocally confirmed the advantages of the new lumefantrine formulation [1]. While the dependency on fat contents is totally removed, the pH dependency has also been optimized such that the new lumefantrine formulation would be solubilized across the expected pH ranges of 1.2 to 6.8 in the human gastrointestinal tract [1].

However, there is one important missing link in the current research that would have further enhanced the utility of the work. The lack of in vivo pharmacokinetic study brings in a void although one may justify that in vitro performance of the new lumefantrine formulation is a good surrogate of its in vivo pharmacokinetics and exposure in humans. It is acknowledged that it may be difficult to perform a human clinical study because of plethora of reasons, however, one should at least consider performing a non-clinical study (say in dogs), which is geared towards establishing the in vivo pharmacokinetics of the new lumefantrine formulation in comparison with the marketed formulation. Since the ultimate goal of developing a new lumefantrine formulation is to enable a consistent and controlled delivery of lumefantrine in patients with malaria, such in vivo pharmacokinetic data would have supported the true utility of the new formulation vs the current marketed formulation. Another important reason to do an in vivo pharmacokinetic study stems from the fact that this class of compounds are prone to be absorbed through lymphatic transport. In this context, lymphatic transport of halofantrine, another anti-malarial drug, has been well established [3,4]. Hence, one key question that would remain unanswered would be: does the new lumefantrine self nanoemulsifying formulation be viable for lymphatic transport mechanism? and/or due to change in its composition and avoidance of food intake, would it circumvent lymphatic transport mechanism?
